# Meta-Analysis of Differential miRNA Expression after Bariatric Surgery

**DOI:** 10.3390/jcm8081220

**Published:** 2019-08-15

**Authors:** Gladys Langi, Lukasz Szczerbinski, Adam Kretowski

**Affiliations:** 1 Clinical Research Centre, Medical University of Bialystok, 15-276 Bialystok, Poland; 2 Department of Endocrinology, Diabetology and Internal Medicine, Medical University of Bialystok, 15-276 Bialystok, Poland

**Keywords:** microRNA, bariatric surgery, Type 2 Diabetes, obesity

## Abstract

Bariatric surgery is an efficient treatment for weight loss in obese patients and for resolving obesity comorbidities. However, the mechanisms behind these outcomes are unclear. Recent studies have indicated significant alterations in the transcriptome after surgery, specifically in the differential expression of microRNAs. In order to summarize the recent findings, we conducted a systematic summary of studies comparing microRNA expression levels before and after surgery. We identified 17 animal model and human studies from four databases (Ovid, Scopus, Web of Science, and PubMed) to be enrolled in this meta-analysis. From these studies, we identified 14 miRNAs which had the same direction of modulation of their expression after surgery in at least two studies (downregulated: hsa-miR-93-5p, hsa-miR-106b-5p, hsa-let-7b-5p, hsa-let-7i-5p, hsa-miR-16-5p, hsa-miR-19b-3p, hsa-miR-92a-3p, hsa-miR-222-3p, hsa-miR-142-3p, hsa-miR-140-5p, hsa-miR-155-5p, rno-miR-320-3p; upregulated: hsa-miR-7-5p, hsa-miR-320c). Pathway analysis for these miRNAs was done using database resources (DIANA-TarBase and KEGG pathway database) and their predicted target genes were discussed in relation with obesity and its comorbidities. Discrepancies in study design, such as miRNA source, bariatric surgery type, time of observation after surgery, and miRNA profiling methods, were also discussed.

## 1. Introduction

Bariatric surgery was first performed in 1963 to help obese patients lose excess weight permanently [1]. Since then, numerous surgery procedures were developed with varying gastrointestinal effects [1,2]. For example, Sleeve Gastrectomy (SG) and Gastric Band are primarily restrictive to limit food intake and induce early satiety, while Roux-en-Y (RYGB) is both restrictive and malabsorptive [1,2]. All procedures result in significant weight loss (14.9%–28.4%) and minimal weight regain (1.4%–3.9%) years after surgery [3,4,5]. SG is more popular in Poland and the US [6,7,8] as it is a relatively less complicated procedure and has less surgery complications and reoperation compared to other procedures [4,9,10,11,12,13,14].

In addition to weight loss, many bariatric surgery patients demonstrate improvement in comorbidities of obesity post-operation. This includes recovery from Type 2 Diabetes Mellitus (T2DM) and achieving long-term favorable levels of cardiovascular risk factors, such as high-density lipoprotein cholesterol and hypertension [4,15,16]. Bariatric surgery is also associated with reduced risk of obesity-related cancers, such as colon and endometrial cancer [17]. Although this surgery is mainly reserved for class III obese patients (BMI > 40 kg/m^2^), it is also recommended for less obese patients (BMI > 35 kg/m^2^) with obesity comorbidities due to these beneficial outcomes [16,18].

In the case of T2DM, glucose control via surgical treatment was reported to be better than medical therapy [19,20,21,22]. Recently, bariatric surgery is endorsed as a treatment for obese diabetic patients by the International Diabetes Federation, American Diabetes Association (ADA), and American College of Surgeons [23]. However, T2DM remission rates appear to differ between surgery procedures, where RYGB has higher rates compared to SG and Gastric Band [4,24]. A study reported 60.2% of RYGB patients achieved diabetes remission after 7 years of surgery, compared to 20.3% for Gastric Banding [4]. Long-term diabetes remission for SG was quite low in two studies: 35.3% for Taiwanese patients and 28% for American patients after 5 years of surgery [13,24], but another study reported a remission rate of 66% five years after SG [25]. 

The mechanisms of these long-term beneficial effects after bariatric surgery is poorly understood. The acronym “BRAVE” is often used to describe RYGB physiological effects, which are to alter bile flow, restrict stomach size, alter anatomy/flow of nutrients, manipulate vagal, and modulate enteric gut and adipose hormones [26]. However, these effects cannot explain all the observed metabolic changes associated with RYGB [27]. Thus, researchers are looking into the molecular biological explanations for these metabolic effects after surgery. Novel biomarkers from these studies would not only help us understand the mechanisms behind bariatric surgery outcomes, but also serve as patient-level factors for predicting these outcomes [2].

Epigenetic changes due to surgery could give insight into these mechanisms. Epigenetic machinery, such as DNA methylation, histone modifications, and non-coding RNAs, can respond to external environmental cues by altering gene expression levels without changing DNA sequence. In recent years, there is an increasing interest in studying the relationship between epigenetic changes and bariatric surgery outcomes [28]. Among them are studies on microRNAs (miRNA) [29,30,31,32,33,34,35,36,37,38,39,40,41,42,43,44,45]. These small non-coding RNAs (21–22 nucleotides) are important for regulating gene expression post-transcriptionally. Single-stranded miRNA binds to a complementary target messenger RNA (mRNA) to disrupt translational processes [46,47,48,49]. A single miRNA can have multiple targets and regulate many different biological pathways [50,51,52]. Studies have reported miRNAs that regulate obesity-related pathways [53,54,55,56,57,58,59,60] and miRNA dysregulation is linked to obesity and its comorbidities [29,61,62,63,64].

Several miRNA studies have reported short- and long-term miRNA profile changes after bariatric surgery in various tissues of animal models and humans [29,30,31,32,33,34,35,36,37,38,39,40,41,42,43,44,45]. However, these studies typically have small sample sizes, use different profiling strategies, and study different types of bariatric surgery. There are no literature reviews so far on these surgery-related miRNAs. Thus, this study aims to identify consistently modulated miRNAs after bariatric surgery and report biological pathways that are predicted to be regulated by these miRNAs. These pathways may give insight into the molecular mechanisms behind weight loss and remission of obesity comorbidities after bariatric surgery.

## 2. Methods

### 2.1. Search Strategies

The databases for the literature search were chosen based on a recommendation of the optimal database combinations [65] and database accessibility in our institution. The four databases chosen were Ovid, Scopus, Web of Science, and PubMed. The databases were searched for studies profiling modulation of miRNA expression in bariatric surgery patients published up until 10 February 2019. The search terms were: (miRNA AND Bariatric surgery) OR (microRNA AND Bariatric surgery). For Ovid, an advanced search was used with the search terms. A basic search was used for the other databases. 

### 2.2. Study Selection

During the screening stage, the exclusion criteria were: (1) non-English publications, (2) abstracts-only publications, case reports, comments, or reviews, (3) no report or comparison of miRNA profiles before and after surgery or between bariatric surgery-operated animals and sham-operated animals, or (4) added another intervention post-surgery before miRNA assessment. Inclusion criteria were (1) animal and human studies, (2) any profiling method, (3) any bariatric surgery method, (4) any biological sample type, and (5) reported cut-off criteria for differentially expressed miRNAs. One full-text study was later excluded due to inconsistent reporting of the direction of miRNA expression.

### 2.3. Data Collection Process

The items collected from the full text and Supplementary Information followed a recent methods paper for meta-analysis of miRNAs studies [66]. The items were: first author, year of publication, digital object identifier (DOI) when available, study location, species of the samples, tissue types, bariatric surgery type, sample sizes, body mass index (BMI) before and after surgery, comparison groups, number of follow-up visits and their time after surgery, miRNA expression profiling platform, cut-off criteria of dysregulated miRNAs, and the list of differentially expressed miRNAs. Study authors were contacted to identify missing information on bariatric surgery type.

### 2.4. Synthesis of Results

Only miRNAs reported in at least two independent studies were retained for analysis. The selected miRNAs were grouped into three categories based on their consistency. The first group included miRNAs with consistent report of expression direction in two or more studies. The second group included miRNAs with some discrepancies in the direction, but two or more studies agreed on a direction. The third group included miRNAs with no consistent reports of expression direction. Pathway analysis was done only for the first two miRNA groups. Pathway analysis was done using DIANA miRPath v.3 to predict their target genes and KEGG pathways (http://www.microrna.gr/ miRPathv3) [67].

## 3. Results

### 3.1. Selected Studies for the Meta-Analysis

**Figure 1:**
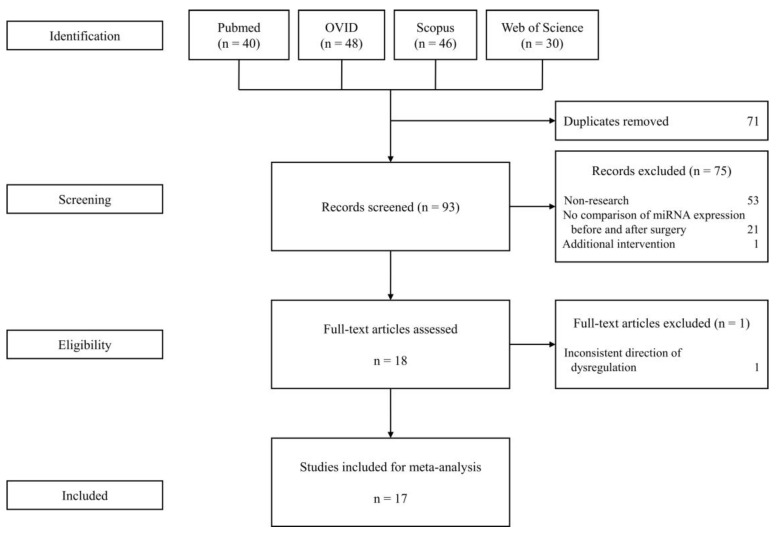
Flow diagram for study selection.

A total of 164 articles were retrieved from Pubmed, OVID, Scopus, and Web of Science. After screening and assessment, 17 studies were selected for the meta-analysis (Figure 1). These studies have varying sources of miRNA, surgery type, and profiling strategies.

Most reported studies profiled miRNA levels before and after bariatric surgery in human patients (*n* = 13) (Table 1) [29,30,31,32,33,36,37,38,40,41,42,43,44], but some studied animal models (*n* = 4) [34,35,39,45]. The human studies were conducted in Caucasian [29,30,31,33,36,37,38,41,42,43] and Asian populations [32,40,44]. Most of these studies have small sample sizes (less than 30 participants; *n* = 15). However, a recent study in Austria profiled 58 patients [36] and a study in China profiled 124 patients [44]. The human studies mostly had more female patients, with the exception of one study [33], while animal studies investigated exclusively male animals. 

The studies isolated miRNAs from different tissues: blood (plasma and serum) (*n* = 7) [29,30,31,33,36,40,45], circulating exosomes [32,37], monocytes [38], circulating endothelial progenitor cells [44], adipose tissue [41,42,43], liver [34,35,45], and hypothalamus [39] (Table 2).

The studies also differ in the miRNA profiling strategies (Table 2). For isolation methods, the studies used either mirVANA isolation kits (*n* = 5), miRNeasy kits (*n* = 5), TRIzol reagent (*n* = 3) or other kits. Most of the studies then used qPCR (*n* = 7) or microarrays (*n* = 5) as their main profiling method. RNA sequencing was used as the main analysis in one study [32]. Other studies (*n* = 4) used a screening step using high-throughput profiling methods, such as microarrays and RNA sequencing, in a subset of their patients, then qPCR as validation in the final sample. Human studies using microarrays used the Robust MultiArray Average (RMA) method for normalization (*n* = 3). Studies with cells and tissue samples normalized their data using small non-coding RNAs with RNU6 and RNU6B being most commonly used (*n* = 6). Studies with plasma and serum samples used a number of stable miRNAs, which were unique for each study. One plasma study used RNA spike-in levels for normalization [36] and the RNA sequencing study used DESeq2 package for normalization [32].

The surgery type most commonly assessed is RYGB (*n* = 13) (Table 3) [29,30,31,32,36,37,38,39,40,41,42,43,45], but one study collected two SG patients in addition to RYGB [32] and one study profiled only SG patients [33]. In rats, the studies compared a duodeno–jejunal bypass (DJB) [34,35] or RYGB [39,45] with sham surgery. One study also performed SG in rats to compare with DJB results [34].

Lastly, the studies differ in the duration of study and number of observations after bariatric surgery (Table 3). One study of RYGB patients profiled miRNAs in five time points (1-, 3-, 6-, 9-, and 12-months post-surgery) [30]. Two studies in rats also studied miRNA levels two-, four-, and eight-weeks post-surgery [34,35]. Other studies only profiled miRNA once after surgery. Time of observation also differs between studies. Some studies looked into short-term expression changes (less than or equal to 3 months; *n* = 9), while others looked at long-term response (*n* = 9; maximum 2-years post-surgery).

### 3.2. Differential Expression of miRNA before and after Surgery

According to the selected studies, a total of 50 miRNA families and 205 unique miRNAs were significantly differentially expressed after surgery compared to baseline. Among these, 32 differentially expressed miRNAs were identified in at least two different studies. The 32 miRNAs can be grouped based on the consistency of findings and reasons for discrepancies (Table 4).

Group 1 includes 14 miRNAs that changed in the same direction of expression, regardless of sample type and time of observation (downregulated: hsa-miR-93-5p, hsa-miR-106b-5p, hsa-let-7b-5p, hsa-let-7i-5p, hsa-miR-16-5p, hsa-miR-19b-3p, hsa-miR-92a-3p, hsa-miR-222-3p, hsa-miR-142-3p, hsa-miR-140-5p, hsa-miR-155-5p, rno-miR-320-3p; upregulated: hsa-miR-7-5p, hsa-miR-320c). Group 2 includes six miRNAs with inconsistent findings, but at least two studies agreed on a direction of expression (overall downregulated: hsa-miR-125b-5p, hsa-miR-130-3p, hsa-miR-221-3p, hsa-miR-146a-5p, rno-miR-122-5p; overall upregulated: rno-miR-503-5p). For example, hsa-miR-125b-5p was found to be downregulated in two studies profiling miRNA from plasma samples, but was upregulated in an exosome study. Lastly, group 3 includes 12 miRNAs reported in two studies but with no agreement in direction (hsa-miR-21-5p, hsa-miR-33a-5p, hsa-miR-320a-3p, hsa-miR-320b, hsa-miR-378a-3p, hsa-miR-103-3p, rno-miR-133b-3p, rno-miR-194-5p, hsa-miR-122-5p, rno-miR-146a-5p, rno-miR-542-3p, hsa-miR-191-5p).

### 3.3. Pathway Analysis

**Figure 2:**
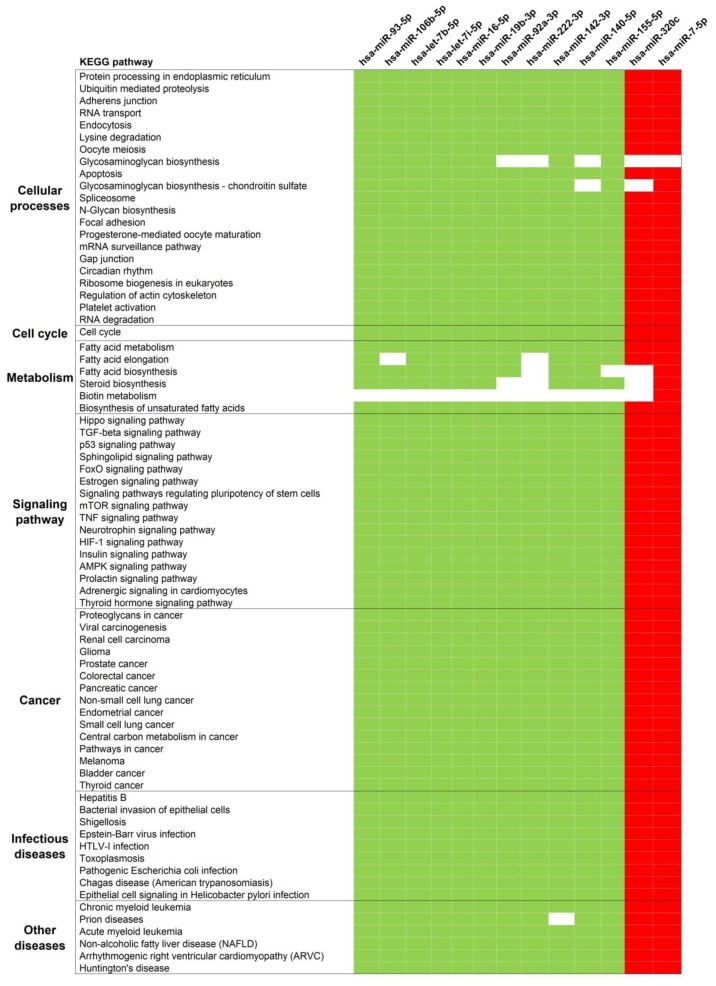
Significantly enriched KEGG pathways of surgery-responsive miRNAs. The miRNAs reported to be involved in a particular pathway are indicated in colors green or red; otherwise, they are indicated as white. Green indicates pathways targeted by down-regulated miRNAs. Red indicates pathways targeted by up-regulated miRNAs.

DIANA-miRPath was used to identify pathways regulated by miRNAs in Group 1 and 2. The first analysis was done with only Group 1, and a total of 74 KEGG pathways were significantly predicted to be regulated by these miRNAs. The miRNAs were predicted to target genes involved in cancer, cell cycle, fatty acid metabolism, signaling pathways, infectious diseases, and RNA processes in cells (Figure 2). The inclusion of Group 2 miRNAs resulted in a slightly different pathway profile. This secondary analysis retained most pathways from the first analysis (69 out of 74) and added eight different significant pathways. The additional pathways were related to signaling pathways, metabolism, and biosynthesis processes (not shown).

## 4. Discussion

The benefits of bariatric surgery beyond weight loss, such as T2DM remission, have been reported extensively [15,16,19,20,21,22]. However, the mechanisms behind successful weight loss and improvement of obesity comorbidities are poorly understood. In recent years, more and more studies are looking into a patient’s miRNAome before and after bariatric surgery. The miRNA profile changes as a response to environmental changes, including bariatric surgery. Understanding how miRNA profile changes due to bariatric surgery might uncover important pathways behind its outcomes.

We found that through February 2019, there were 17 studies on miRNA profiles of patients before and after bariatric surgery. Although a relatively small number, there is a sharp increase in publications in the last five years. The first study among them was published in 2012 [38] and 15 studies were published in and after 2015. This indicates a rapid increase in interest of miRNAs related to bariatric surgery. These studies consistently found differential expression of miRNAs after surgery in various tissues with a total of 205 unique miRNAs reported so far. This is in contrast to other genetic studies that found inconsistent findings of the influence of bariatric surgery on DNA methylation [28,69] and no associations between Single-Nucleotide Polymorphisms with weight loss success after bariatric surgery [70,71].

However, these recent miRNA studies were highly variable in study design. Studies on rats looked into a wide range of tissues and included tissues inaccessible in human studies, such as the hypothalamus and liver. Most human studies profiled easily accessible tissues, including circulating miRNA in plasma, serum, exosomes, and monocytes. Some studies had access to adipose tissue biopsies which were collected from patients a few years after surgery. In contrast, human blood samples were able to be collected earlier and at more time points. The earliest time point was 21 days after surgery [31] and one study had five time points after surgery [30]. The sample type and time of observation appeared to be the main reasons for the discrepancy in miRNA expression direction, especially in Group 3’s miRNAs. For example, hsa-mir-21-5p, hsa-miR-320a-3p, hsa-miR-320b, and hsa-miR-378a-3p expressions appear to be time-dependent. Whereas, hsa-miR-33a-5p appears to have sample-specific expression, where its expression was increased in plasma samples, but reduced in exosomes. The other seven miRNAs in Group 3 had both sample type and time differences between the studies that reported them. Studies with more participants on the same sample type and time points are needed to confirm the time and tissue specificity of these miRNAs.

Despite the high variability between studies, there were 14 human and rat miRNAs with consistent direction of differential expression after surgery. In at least two studies, hsa-miR-93-5p, hsa-miR-106b-5p, hsa-let-7b-5p, hsa-let-7i-5p, hsa-miR-16-5p, hsa-miR-19b-3p, hsa-miR-92a-3p, hsa-miR-222-3p, hsa-miR-142-3p, hsa-miR-140-5p, hsa-miR-155-5p, and rno-miR-320-3p were reported to have lower expression levels, while hsa-miR-7-5p and hsa-miR-320c had increased expression levels after surgery. These miRNAs are predicted to be important in various cellular pathways, including those related to lipid metabolism, insulin signaling pathway, and cardiac function. The genes within these pathways are interesting targets for functional studies to understand the mechanisms behind weight loss and remission of obesity-related comorbidity after surgery.

For instance, the most significant pathway is the “proteoglycans in cancer” (hsa05205) and the 13 human miRNAs were predicted to target 140 genes in this pathway. One of them is FZD7, which is one of the Frizzled (Fzd) transmembrane receptors for Wnt proteins [72]. Reduced expression of Wnt proteins is associated with obesity [73]. The hsa-miR-142-3p, which was reported to be downregulated after surgery, is predicted to interact with FZD7. This might lead to an increase in FZD7 expression, activation of the Wnt/Fzd signaling, and thus attenuation of obesity.

These miRNAs were also predicted to target 30 genes in the fatty acid metabolism pathway. The upregulated hsa-miR-7-5p was predicted to target FASN, which is inversely correlated with parameters of glycemic status [74] and its expression is elevated in numerous obesity-related cancers [75]. The downregulation of FASN would result in lower risks for these comorbidities. In addition, Ortega et al. focused on inflammation-responsive miRNAs in adipose tissues [43] and among them, hsa-miR-155-5p and hsa-miR-222-3p were included in the Group 1 miRNAs. The hsa-miR-155-5p has been reported to be elevated in numerous inflammatory conditions [76]. Transfection of an hsa-miR-155 inhibitor in myeloid cells was found to decrease proinflammatory cytokine expression [77]. Deregulation of hsa-miR-155-5p and hsa-miR-222 was also found to be associated with cardiovascular diseases [78,79]. These reports indicate that these miRNAs might be involved in the mechanisms behind reduced inflammation and cardiovascular risks after bariatric surgery. Functional studies are needed to determine the role of these surgery-responsive miRNAs in promoting bariatric surgery outcomes.

Although limited in sample size and the number of miRNAs analyzed, studies on SG patients and animal models suggest different miRNA profiles compared to other surgery types. A study in rats compared rno-miR-200a-3p expression levels between DJB and SG [34]. In this study, rno-miR-200a-3p expression was significantly higher in DJB compared to sham-operated animals. In contrast, this miRNA expression was unchanged after SG and comparable to the sham-operated group [34]. In humans, a study of SG patients reported significant increase in hsa-miR-122-5p levels in serum after surgery [33], but another study reported decreased levels of hsa-miR-122-5p in plasma after RYGB [29]. The discrepancy might explain the apparent differences in bariatric success rates between RYGB and SG, especially concerning the remission of comorbidities such as diabetes. More comparative studies between RYGB and SG patients are needed to confirm these observations. 

However, it is interesting that although many studies used high-throughput methods, only 32 miRNAs were reported in at least two studies. This might be due to the differences in miRNA isolation, profiling, and normalization strategies between studies. For isolation methods, some studies showed that miRNeasy isolation kits produce higher RNA quantity and better quality compared to miRVana [80,81]. Maximizing the isolated miRNA yield is particularly important for plasma and serum samples as their miRNA abundance is significantly lower than tissues [80]. Low yield might result in failure of detecting low-abundance miRNAs [80] and this may contribute to the poor agreement in miRNA profiles between plasma and serum studies [82].

The highly different profiling methods between studies could also be the source of this limited agreement in their findings. Comparative studies have found low correlation between different profiling methods when used to analyze the same samples [82]. Different microarray platforms were found to share a large number of common miRNAs, but the vast majority of the differentially expressed calls were not unanimous across platforms [83]. The median rank correlation between microarray platforms in a different study was only 0.55, while the median correlation between microarray and qPCR was 0.7 [84]. However, one microarray platform had a correlation of lower than 0.5 with qPCR [84]. The cause of this disagreement is unclear [83]. For different qPCR-based platforms, a study found good correlation of CT data between two platforms, but gel electrophoresis suggests a large number of false positive results for an assay [82]. Although these comparative studies did not compare the exact arrays used in our analysis, they suggest there might also be little agreement between profiling methods in our selected studies, leading to a limited number of miRNAs reported in two or more studies. 

Finally, normalization is crucial for providing robust expression data, but there is no consensus regarding normalization methods for miRNA results [85]. Several studies have discussed commonly used normalization methods and found that small nuclear RNAs such as U6 are not good normalizers for miRNA expression [82,85]. This is because RNU6 and other small nuclear RNAs do not reflect the biochemical character of miRNAs and their efficiency throughout the profiling experiments may differ from miRNAs [82,85]. However, many studies profiling miRNA from bariatric surgery patients used RNU6 as their normalization method. Several authors recommend a global mean normalization of a set of reference genes, which may be tissue-specific, with a minimum of three stable housekeeping genes [82,86]. Some of the studies in our analysis used this method, particularly studies with plasma and serum samples.

In addition to these study design limitations, our analysis has not considered population and sex differences, as well as analyzing miRNA results by sample type due to the limited number of studies published so far. Some studies have demonstrated population-specific miRNA expression between populations [87,88]. For example, a study found 16% of the evaluated miRNAs differ significantly between these Caucasians and Africans [87]. There were three studies in Asians in this analysis and their miRNA modulation patterns might not be the same as those of Caucasians. More studies with Asians and other populations should be done to investigate population-specific patterns in miRNA modulation after surgery. Sex differences were also not explored in the current analysis as most human studies were carried out in female patients, while animal studies were performed in male rats. Recent studies in patients and healthy participants have reported sex-biased miRNA expression [89,90]. More studies with male patients are needed to investigate sex-biased miRNA patterns after bariatric surgery. Lastly, our analysis combined findings from different tissue types, but this global approach might mask tissue-specific miRNA patterns after surgery. Unfortunately, there are limited human studies comparing miRNA profiles in tissue samples before and after surgery. As mentioned before, this is likely because of the difficulty in obtaining tissues after surgery. The three studies using SAT samples collected the tissues from the same hospital in Spain [41,42,43]. Only two miRNAs (hsa-miR-155-5p and hsa-miR-221-3p) were reported in at least two of these studies. This is because two SAT studies had targeted miRNA profiling, where Mysore et al. profiled only hsa-miR-221-3p and Ortega et al. profiled only inflammation-induced miRNAs in one study [43]. More untargeted miRNA studies from SAT samples are needed to explore tissue-specific miRNA patterns after surgery. 

## 5. Conclusions

We have identified 14 miRNAs with consistently altered expression after bariatric surgery, regardless of sample type, surgery type, and time of observation after surgery. However, these findings should be taken with caution. These miRNAs were identified from 13 studies with highly variable study design and small sample sizes. A consensus in miRNA profiling methods is crucial for a better comparative study of profiling studies. Until then, a better analysis would be to compare findings of studies with similar strategies. Future studies should also aim to profile a larger number of participants and untargeted profiling of SAT samples. Additionally, more profiling studies in different populations and in males are needed to investigate the generalizability of miRNA modulation after surgery. Studies investigating SG patients are also needed as this surgery type is becoming the most commonly used technique in many countries. Finally, functional studies are needed to understand the role of these miRNAs in promoting weight-loss and remission of obesity-related comorbidities after bariatric surgery. This may lead to novel targets for non-surgical treatment of obesity and its comorbidities and provide novel biomarkers for predicting bariatric surgery outcomes.

## Figures and Tables

**Table 1:** The studies selected for meta-analysis.

Study	Year	Country	Sample Size	Sex (Males/Females)
**Human studies (comparing before vs. after bariatric surgery)**				
Ortega et al. [29]	2013	Spain	22	5/17
Alkandari et al. [30]	2018	UK	9	4/5
Atkin et al. [31]	2019	USA	29	9/20
Bae et al. [32]	2019	South Korea	12	Unspecified
Blum et al. [33]	2017	Israel	21	14/7
Hohensinner et al. [36]	2018	Austria	58	17/41
Hubal et al. [37]	2017	USA	6	0/6
Hulsmans et al. [38]	2012	Belgium	21	7/14
Lirun et al. [40]	2015	China	18	4/11
Mysore et al. [41]	2017	Spain	22	0/22
Ortega et al. [42]	2015	Spain	25	0/25
Ortega et al. [43]	2015	Spain	9	0/9
Wang et al. [44]	2018	China	124	46/78
**Animal studies (comparing bariatric vs sham surgery)**				
Guo et al. [34]	2016	China	35	35/0
Wei et al. [35]	2018	China	45	45/0
Kwon et al. [39]	2015	South Korea	25	25/0
Wu et al. [45]	2015	UK	12	12/0

**Table 2:** Tissue source and miRNA profiling strategies.

Study	Tissue	Isolation	Platform	Normalization
**Human studies**				
Ortega et al. [29]	Plasma	mirVana PARIS Isolation Kit	TaqMan array miRNA cards in a subset and qPCR in the final sample	Geometric mean of six miRNAs (hsa-miR-106a-5p, hsa-miR-146a-5p, hsa-miR-19b-3p, hsa-miR-223-3p, hsa-miR-186-5p, hsa-miR-199a-3p)
Alkandari et al. [30]	Plasma	mirVana PARIS Isolation Kit	miRCURY qPCR panel	Four miRNAs (hsa-miR-223-3p, hsa-miR-26a-5p, hsa-miR-101-3p, and hsa-miR-19a-3p)
Atkin et al. [31]	Plasma	miRCURY RNA Isolation kit	qPCR and a FANTOM miRNA atlas [68]	Global mean
Bae et al. [32]	Exosome	miRNeasy Mini Kit	Small RNA sequencing	Relative log expression using DESeq2
Blum et al. [33]	Serum	miRNeasy serum/plasma kit	RNA sequencing in a subset and qPCR in the final sample	hsa-miR-451a
Hohensinner et al. [36]	Plasma	miRNA tissue lysis kit	qPCR	RNA spike-in
Hubal et al. [37]	Exosome	mirVANA miRNA Isolation Kit	GeneChip miRNA 4.0 Array	RMA algorithm
Hulsmans et al. [38]	Monocytes	TRIzol reagent	qPCR	RNU5G
Lirun et al. [40]	Plasma	mirVana RNA Isolation Kit	GeneChip miRNA 3.0 Array	RMA algorithm
Mysore et al. [41]	Subcutaneous Adipose Tissue (SAT)	miRNeasy Mini kit	qPCR	RNU44
Ortega et al. [42]	SAT	miRNeasy Mini Kit	GeneChip miRNA 3.0 array in a subset and qPCR in the final sample	RMA algorithm and RNU48
Ortega et al. [43]	SAT	miRNeasy Mini Kit	qPCR	RNU6B
Wang et al. [44]	Circulating Endothelial Progenitor Cells	High Pure RNA kit	qPCR	RNU6
**Animal studies**				
Guo et al. [34]	Liver	TRIzol reagent	miProfile Customized Rat qPCR arrays	5S rRNA and RsnRNA U6
Wei et al. [35]	Liver	TRIzol reagent	miProfile Customized Rat qPCR arrays	5S rRNA, RsnRNA U6, rno-miR-25, and rno-miR-186
Kwon et al. [39]	Hypothalamus, Heart, and Liver	Unspecified	Agilent Rat miRNA 8x15k microarray for hypothalamus and heart samples, then qPCR for liver and validation	Whole-array and RNU6
Wu et al. [45]	Plasma and Liver	mirVANA PARIS RNA Isolation kit	TaqMan Array Rodent Card	RNU6-1, RNU6-2, rno-miR-16-5p, rno-miR-223-3p, mmu-miR-1937b

**Table 3:** Bariatric surgery type and time of observation after surgery.

Study	Year	Bariatric Surgery Type	Time of Observation after Surgery
**Human studies**			
Ortega et al. [29]	2013	RYGB	12 months
Alkandari et al. [30]	2018	RYGB	1, 3, 6, 9, and 12 months
Atkin et al. [31]	2019	RYGB	21 days
Bae et al. [32]	2019	RYGB and SG	6 months
Blum et al. [33]	2017	SG	3 months
Hohensinner et al. [36]	2018	RYGB	24 months
Hubal et al. [37]	2017	RYGB	12 months
Hulsmans et al. [38]	2012	RYGB	3 months
Lirun et al. [40]	2015	RYGB	3 months
Mysore et al. [41]	2017	RYGB	24 months
Ortega et al. [42]	2015	RYGB	24 months
Ortega et al. [43]	2015	RYGB	24 months
Wang et al. [44]	2018	Not specified	3 months
**Animal studies**			
Guo et al. [34]	2016	DJB and SG	2, 4, 8 weeks
Wei et al. [35]	2018	DJB	2, 4, 8 weeks
Kwon et al. [39]	2015	RYGB	25 days
Wu et al. [45]	2015	RYGB	53 days

**Table 4:** Differentially expressed miRNA before vs after surgery reported in at least two studies.

	miRNA	miRBase	References	Direction of Expression	No. of Subjects	Tissue	Time of Observation
	**Group 1 miRNAs (same direction of expression after surgery in two or more studies)**						
1	hsa-miR-93-5p	MIMAT0000093	Lirun [40]	−	15	Plasma	3 months
			Alkandari [30]		9	Plasma	3 months
2	hsa-miR-106b-5p	MIMAT0000680	Lirun [40]	−	15	Plasma	3 months
			Alkandari [30]		9	Plasma	3, 12 months
3	hsa-let-7b-5p	MIMAT0000063	Lirun [40]	−	15	Plasma	3 months
			Alkandari [30]		9	Plasma	3 months
4	hsa-let-7i-5p	MIMAT0000415	Lirun [40]	−	15	Plasma	3 months
			Alkandari [30]		9	Plasma	6, 9 months
			Atkin [31]		29	Plasma	21 days
5	hsa-miR-16-5p	MIMAT0000069	Lirun [40]	−	15	Plasma	3 months
			Hubal [37]		6	Exosomes	12 months
6	hsa-miR-19b-3p	MIMAT0000074	Ortega [43]	−	9	SAT	24 months
			Lirun [40]		15	Plasma	3 months
			Ortega [29]		22	Plasma	12 months
7	hsa-miR-92a-3p	MIMAT0000092	Lirun [40]	−	15	Plasma	3 months
			Alkandari [30]		9	Plasma	9, 12 months
8	hsa-miR-222-3p	MIMAT0000279	Ortega [29]	−	22	Plasma	12 months
			Ortega [43]		9	SAT	24 months
9	hsa-miR-142-3p	MIMAT0000434	Bae [32]	−	12	Exosome	6 months
			Ortega [29]		22	Plasma	12 months
10	hsa-miR-140-5p	MIMAT0000431	Bae [32]	−	12	Exosome	6 months
			Ortega [29]		22	Plasma	12 months
11	hsa-miR-155-5p	MIMAT0000646	Ortega [43]	−	9	SAT	24 months
			Ortega [42]		25	SAT	24 months
12	rno-miR-320-3p	MIMAT0000903	Wu [45]	−	4	Plasma	53 days
			Wei [35]		5	liver	2 months
13	hsa-miR-320c	MIMAT0005793	Atkin [31]	+	29	Plasma	21 days
			Lirun [40]		15	Plasma	3 months
14	hsa-miR-7-5p	MIMAT0000252	Atkin [31]	+	29	Plasma	21 days
			Bae [32]		12	Exosome	6 months
	**Group 2 miRNAs (overall same direction of expression after surgery in two or more studies)**						
1	hsa-miR-125b-5p	MIMAT0000423	Ortega [29]	−	22	Plasma	12 months
			Alkandari [30]	−	9	Plasma	6, 9, 12 months
			Hubal [37]	+	6	Exosomes	12 months
2	hsa-miR-130b-3p	MIMAT0000691	Ortega [42]	−	25	SAT	24 months
			Alkandari [30]	−	9	Plasma	12 months
			Ortega [29]	+	22	Plasma	12 months
3	hsa-miR-221-3p	MIMAT0000278	Ortega [43]	−	9	SAT	24 months
			Ortega [42]	−	25	SAT	24 months
			Mysore [41]	−	22	SAT	24 months
			Lirun [40]	−	15	Plasma	3 months
			Ortega [29]	+	22	Plasma	12 months
4	rno-miR-122-5p	MIMAT0000827	Kwon [39]	−	25	heart	25 days
			Kwon [39]	−	25	liver	25 days
			Wu [45]	−	4	Plasma	53 days
			Wu [45]	−	8	Liver	53 days
			Kwon [39]	+	25	hypothalamus	25 days
5	hsa-miR-146a-5p	MIMAT0000449	Lirun [40]	−	15	Plasma	3 months
			Ortega [43]	−	9	SAT	24 months
			Ortega [29]	+	22	Plasma	12 months
6	rno-miR-503-5p	MIMAT0003213	Kwon [39]	+	25	hypothalamus	25 days
			Kwon [39]	+	25	heart	25 days
			Wei [35]	−	4	liver	2 months
	**Group 3 miRNAs (reported in at least two studies, but with no agreement in direction of expression)**						
1	hsa-miR-21-5p	MIMAT0000076	Alkandari [30]	−	9	Plasma	9, 12 months
			Ortega [29]	+	22	Plasma	12 months
2	hsa-miR-33a-5p	MIMAT0000091	Bae [32]	−	12	Exosome	6 months
			Alkandari [30]	+	9	Plasma	6 months
3	hsa-miR-320a-3p	MIMAT0000510	Alkandari [30]	−	9	Plasma	6, 9, 12 months
			Lirun [40]	+	15	Plasma	3 months
4	hsa-miR-320b	MIMAT0005792	Alkandari [30]	−	9	Plasma	9 months
			Lirun [40]	+	15	Plasma	3 months
5	hsa-miR-378a-3p	MIMAT0000732	Alkandari [30]	−	9	Plasma	6, 9, 12 months
			Lirun [40]	+	15	Plasma	3 months
6	hsa-miR-103-3p	MIMAT0000101	Lirun [40]	−	15	Plasma	3 months
			Hubal [37]	+	6	Exosomes	12 months
7	rno-miR-133b-3p	MIMAT0003126	Wei [35]	−	4	liver	2 months
			Kwon [39]	+	25	hypothalamus	25 days
8	rno-miR-194-5p	MIMAT0000869	Kwon [39]	−	25	heart	25 days
			Guo [34]	+	4	liver	2 months
9	hsa-miR-122-5p	MIMAT0000421	Ortega [29]	−	22	Plasma	12 months
			Blum [33]	+	21	Serum	3 months
10	rno-miR-146a-5p	MIMAT0000852	Wu [45]	−	4	Plasma	53 days
			Kwon [39]	+	25	hypothalamus	25 days
11	rno-miR-542-3p	MIMAT0003179	Wei [35]	−	4	liver	2 months
			Kwon [39]	+	25	hypothalamus	25 days
12	hsa-miR-191-5p	MIMAT0000440	Lirun [40]	−	15	Plasma	3 months
			Bae [32]	+	12	Exosome	6 months
